# Development of a Highly Efficient Optoelectronic Device Based on CuFeO_2_/CuO/Cu Composite Nanomaterials

**DOI:** 10.3390/ma15196857

**Published:** 2022-10-02

**Authors:** Fatemah H. Alkallas, Amira Ben Gouider Trabelsi, Tahani A. Alrebdi, Ashour M. Ahmed, Mohamed Rabia

**Affiliations:** 1 Department of Physics, College of Science, Princess Nourah bint Abdulrahman University, P.O. Box 84428, Riyadh 11671, Saudi Arabia; 2 Nanophotonics and Applications Lab, Physics Department, Faculty of Science, Beni-Suef University, Beni-Suef 62514, Egypt; 3 Nanomaterials Science Research Laboratory, Chemistry Department, Faculty of Science, Beni-Suef University, Beni-Suef 62514, Egypt

**Keywords:** CuFeO_2_, delafossite, optoelectronic, photoresponsivity, detectivity

## Abstract

Herein, an optoelectronic device synthesized from a CuFeO_2_/CuO/Cu nanocomposite was obtained through the direct combustion of Cu foil coated with Fe_2_O_3_ nanomaterials. The chemical, morphological, and optical properties of the nanocomposite were examined via different techniques, such as XRD, XPS, TEM, SEM, and UV/Vis spectrophotometer. The optical reflectance demonstrated a great enhancement in the CuFeO_2_ optical properties compared to CuO nanomaterials. Such enhancements were clearly distinguished through the bandgap values, which varied between 1.35 and 1.38 eV, respectively. The XRD and XPS analyses confirmed the chemical structure of the prepared materials. The produced current density (J_ph_) was studied in dark and light conditions, thereby confirming the obtained optoelectronic properties. The J_ph_ dependency to monochromatic wavelength was also investigated. The J_ph_ value was equal to 0.033 mA·cm^−2^ at 390 nm, which decreased to 0.031 mA·cm^−2^ at 508 nm, and then increased to 0.0315 mA·cm^−2^ at 636 nm. The light intensity effects were similarly inspected. The J_ph_ values rose when the light intensities were augmented from 25 to 100 mW·cm^−2^ to reach 0.031 and 0.05 mA·cm^−2^, respectively. The photoresponsivity (R) and detectivity (D) values were found at 0.33 mA·W^−1^ and 7.36 × 10^10^ Jones at 390 nm. The produced values confirm the high light sensitivity of the prepared optoelectronic device in a broad optical region covering UV, Vis, and near IR, with high efficiency. Further works are currently being designed to develop a prototype of such an optoelectronic device so that it can be applied in industry.

## 1. Introduction

Light detection through optoelectronic devices has attracted a lot of interest in the last decade, where it covers a wide range of technology instruments, such as cameras, spacecraft, and laser-based rockets. Particularly, optoelectronic devices based on semiconductor materials remain commonly used for these applications [1,2,3]. Their developments facilitate technology advancement, while ensuring cost effective device fabrication. However, light detection highly depends on the wavelengths used and the intensities that control the detection efficiency of the optoelectronic device, which is illustrated through their photoresponsivity and detectivity. Here, the incident photon flux coming from the semiconductor optoelectronic device induces level splitting that forms electron-hole pairs. With an increase in the generated electrons, the produced J_ph_, R, and D values increase, and all the photoreactor efficiencies increase [4,5,6].

Metal oxides are one of the most promising semiconductor materials known, with high stabilities and low-cost properties. These great advantages increase through an increase in the optical behavior, thus, black-color metal oxides have additional photon absorbance behavior and small band gaps. These characteristic properties motivate the application of these oxides in the syntheses of optoelectronic devices [7]. Scientists and researchers are working on raising the additional optical properties of such materials via the enhancement of their surface area and active sites [8,9]. Thus, metal oxide with a large surface becomes the more promising and selected material [10,11,12].

Recently, CuO has gained a lot of interest owing to its many properties, such as its optical absorbance in UV, Vis, near-IR regions, and its small bandgap from 1.2 to 1.5 eV [11,12]. Some studies have been carried out on CuO materials for light detection. Wang et al. [13] synthesized CuO materials as optoelectronic, where the produced J_ph_ value was small (20 µA) at a bias voltage of +5 V. Similarly, Hong et al. [14] studied CuO/Si materials heterostructure as an optoelectronic device with small J_ph_ values of 4.5 µA at zero V. Moreover, Bai et al. [15] studied a ZnO/CuO composite for optoelectronic applications, but the produced J_ph_ values were small at 107 µA at +1 V. Shuai et al. [16] synthesized a CdS-ZnO composite for optoelectronic applications, where the J_ph_ value was 2 µA at 4.5 V.

Delafossite CuFeO_2_ is considered an upcoming and promising P-type material for several applications, such as hydrogen generation, antibacterials, and optoelectronics [17,18,19,20]. This is related to its good optical absorbance behavior and moderate bandgap. CuFeO_2_ has different crystalline natures, such as hexagonal (2H) and rhombohedral 3R, as defined by the layer stacking inside the crystal lattice [21]. Complex synthesis methods have been developed to prepare these materials, such as laser and electrochemical deposition, sputtering, physical deposition, and plasma annealing techniques [22]. CuFeO_2_ can be used as P-type material for different applications, such as electrochemical H2 generation, with a water splitting efficiency of about 3% [23]. Moreover, this material is used in solar energy applications such as solar cells due to its ability to absorb light with high efficiency [24]. Deng et al. synthesized CuFeO_2_ through a sol–gel method to be tested as an optoelectronic; the material appeared responsive to light, indicating good optoelectronic behavior [25]. However, the limited studies carried out on CuFeO_2_ as a potential optoelectronic material show the obtained J_ph_ values were very small.

Herein, a delafossite CuFeO_2_ material was synthesized using a simple and promising technique through the direct combustion of Cu foil coated with Fe_2_O_3_ in air, which led to the formation of CuFeO_2_/CuO/Cu nanomaterials. The chemical analyses: TEM, SEM, XRD, XPS, and UV/Vis spectrophotometer, are used to confirm the morphological and chemical structure, and optical properties. Furthermore, we apply the delafossite CuFeO_2_/CuO/Cu as an optoelectronic material with high photoresponsivity and detectivity results. The optoelectronic study is carried out by testing the effect of light wavelengths (390 to 636 nm) and intensities (25 to 100 mW·cm^−2^) on the optoelectronic device. Moreover, the test is carried out in the dark and under chopped light to confirm the responsivity of the optoelectronic material to light illumination. Finally, a mechanism is detailed explaining the sensitivity of the devices to light illumination.

## 2. Materials and Methods

### 2.1. Materials

FeCl_3_, acetone, and ethanol were purchased from Piochem Company, Cairo, Egypt. Silver paste and Cu foil were purchased from Sigma Aldrich Co., Saint Louis, MO, USA.

### 2.2. Preparation of CuO/Cu

For the preparation of CuO/Cu, the Cu foil (0.3 mm thickness) was ultrasonically cleaned with acetone, ethanol, and distilled water, for 10 min per solution, and then heated in a combustion furnace at 500 °C for 10 min. This process led to the oxidation of the external layer and the formation of a CuO-coated surface.

### 2.3. Preparation of CuFeO_2_/CuO/Cu

An additional step was carried out to prepare CuFeO_2_ on the surface of CuO/Cu. First, Cu foil was dipped inside a (0.1 M) FeCl_3_ solution for 30 min, and then this foil was heated at 70 °C for 1 h. This led to the formation of Fe_2_O_3_ on the surface of the Cu, and then all materials were annealed at 500 °C for 10 min. This ensured the transfer of the upper surface to CuFeO_2_ and the formation of CuFeO_2_/CuO/Cu.

### 2.4. Characterization of CuO and CuFeO_2_/CuO Materials

The crystal structure of the prepared materials was investigated using PANalytical Pro, X-ray diffraction (XRD) (Almelo, The Netherlands). Fourier transform infrared (FTIR), Jasco spectrophotometer, and X-ray photoelectron spectroscopy (XPS) (K-ALPHA, Easton, MA, USA). The morphologies were measured using scanning electron microscopy (SEM) (ZEISS, Oberkochen, Germany), and TEM (JEOL JEM-2100), which was used to determine the morphologies inside the sample. However, the optical spectra were determined through Shimadzu UV/Vis spectrophotometer, Waltham, MA, USA).

### 2.5. The Electrochemical Study

**Figure 1:**
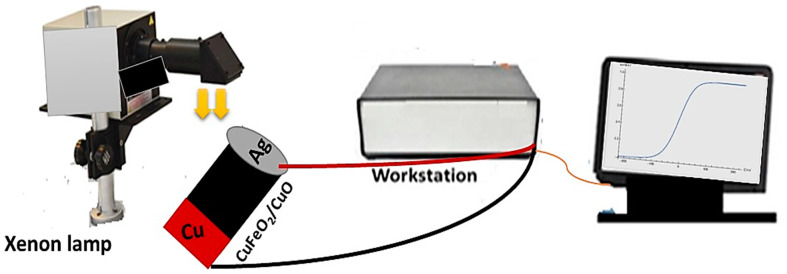
A schematic diagram of the method used to measure the optoelectronic system.

Electrochemical testing was performed via an electrochemical workstation (CHI) from −1.0 to +1.0. The light source was a solar simulator (100 mW·cm^−^^2^). The measurements were carried out under different light wavelengths (from 390 to 636 nm) and different light intensities (from 25 to 100 mW·cm^−^^2^) with a scan rate of 100 mV·s^−^^1^. The prepared photoelectrode had two sides: one side was Cu and the other side was Ag. The Ag paste covered a spot on the optoelectronic film that had contact with the wire of the electrochemical workstation. The effects of light and dark and on/off chopped light were studied. A schematic of the electrochemical measurements can be seen in Figure 1.

## 3. Results and Discussion

### 3.1. Characterization and Analyses

#### The Chemical Structure

**Figure 2:**
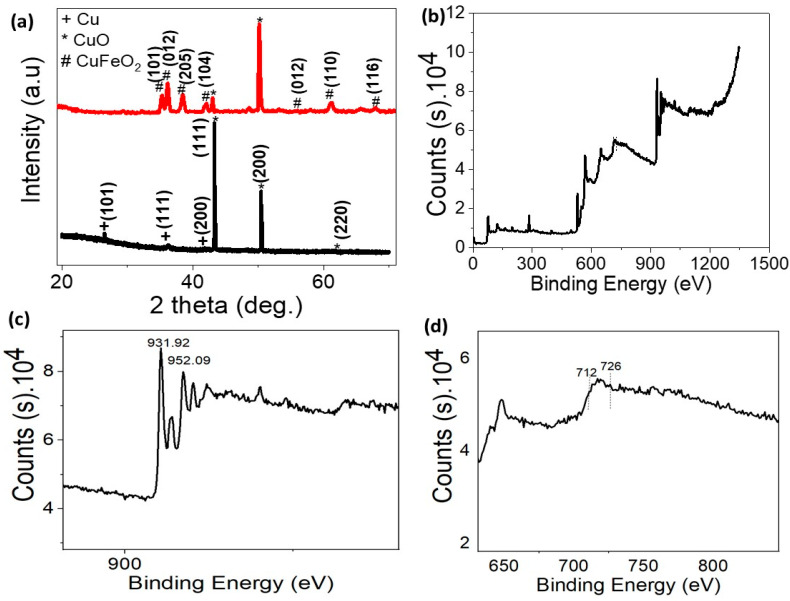
(**a**) The XRD of CuFeO_2_, (**b**) XPS of CuFeO_2_, (**c**) Cu 2P, and (**d**) Fe 2P.

Figure 2a represents the XRD measurements of the synthesized Cu sheets. The associated diffraction illustrates the high crystallinity of the used Cu thin film. Both Cu thin film peaks (111) and (200) were located at 2θ = 43.3° and 50.4°, respectively. The successful deposition of the CuFeO_2_ on Cu/CuO thin film was identified via XRD analysis (see Figure 2a). The main reflection patterns (101), (012), (104), (018), (110), and (116) of a typical CuFeO_2_ structure were located at 2θ = 34°, 35°, 41°, 55°, 61°, and 70°, respectively (JCPDS No. 75-2146). Thus, XRD patterns demonstrated a pure phase of CuFeO_2_ thin film for the current growth condition. This is in accordance with previous studies on CuFeO_2_ thin films. Therefore, XRD measurements demonstrated the synthesis of CuFeO_2_ films with high crystallinity on a Cu/CuO substrate.

Photoelectron spectroscopy (XPS) identified the element composing the grown CuFeO_2_ films (see Figure 2b–d). The XPS survey spectrum of the grown films illustrated the presence of Cu, Fe, O, and C element signatures of single-phase oxide as CuFeO_2_. Indeed, the C_1s_ peak located at 283.92 eV could be associated with the carbon originating from hydrocarbon in ambient air. The Cu_2p_ core level spectrum had two intense peaks at about 952.09 and 931. eV, which corresponds to the Cu _2p1/2_ and Cu _2p3/2_ spin-orbital components. However, the satellite peak of weak intensity illustrated the presence of Cu^2+^ species in the samples. Moreover, Fe 2p XPS demonstrated the well-resolved Fe _2p3/2_ and Fe _2p1/2_ peaks at 712 and 726 eV, as confirmed by earlier studies. The XPS analysis demonstrated that the prepared Cu and Fe in CuFeO_2_ crystals are in +1 and +3 electronic states. The O_1s_ XPS spectra displayed single peaks at 529.08 eV, which was associated with the lattice oxygen in CuFeO_2._ No oxygen defects, i.e., the presence of O^2−^ ions, were located.

**Figure 3:**
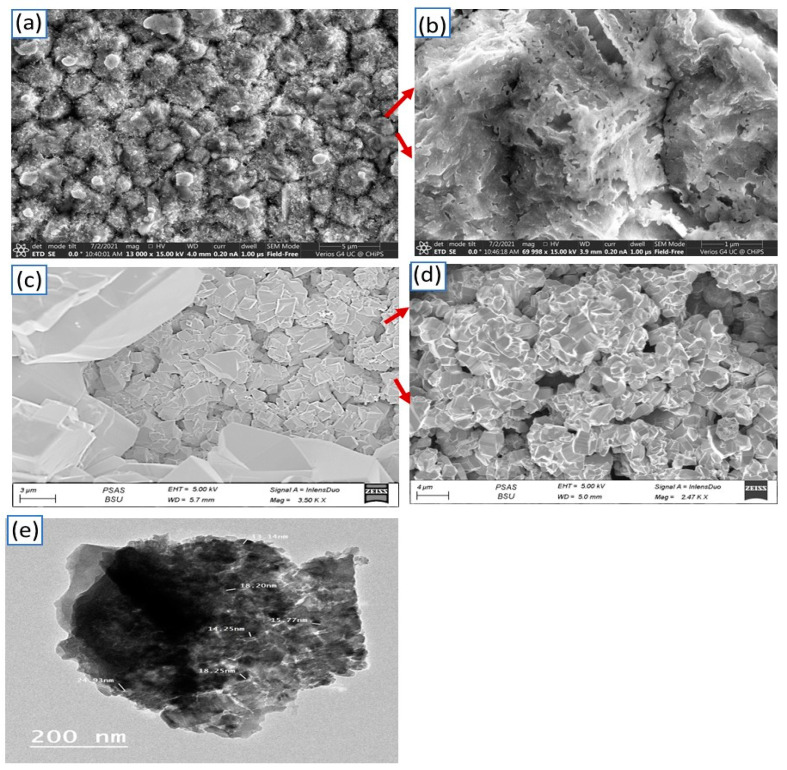
SEM of (**a**,**b**) CuO and (**c**,**d**) CuFeO_2_. (**e**) TEM of CuFeO_2_/CuO.

Figure 3a,b show the SEM images of the CuO films. These figures confirm the formation of porous materials similar to islands. Figure 3b,c show the typical SEM images of the CuFeO_2_ films. Microcrystals were distinguished with rhombohedral morphology, where edges, corners, and surfaces could be identified. The CuFeO_2_ sheet shapes can be located in Figure 3c. Herein, a visible layer’s form was noticed. Particularly, small defects randomly distributed on the surface were observed. This confirms the XPS analysis revealed the presence of defects, which was associated with oxygen defects. The obtained CuFeO_2_ rhombohedra size is captured in the zoomed image in Figure 3d, where a size range varying between 4 and 10 μm was located. TEM images confirm the formation of a porous material with a rhombohedral morphology and crystalline nature, in which the crystal sizes extend from 25 to 200 nm.

**Figure 4:**
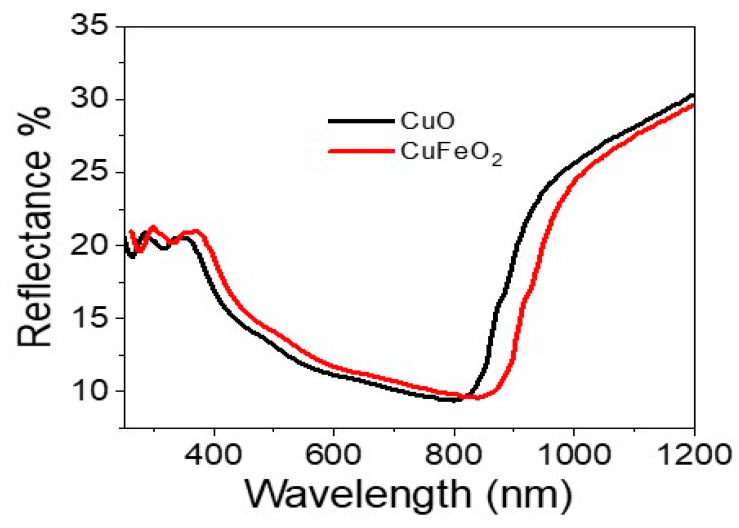
The reflectance of CuO and CuFeO_2_ materials.

The optical reflectance of CuO and CuFeO_2_ materials were determined from the optical characterizations for the samples, as shown in Figure 4. The CuO and CuFeO_2_ materials had almost the same behavior, but the reflectance of CuFeO_2_ had a more enhanced behavior. This indicates the elevated absorbance in UV, Vis, and near-IR regions. The bandgap values were enhanced after the CuFeO_2_ formation, in which CuO and CuFeO_2_ materials had bandgap values of 1.38 and 1.35 eV, respectively. The bandgap values were calculated using Kubelka–Munk equations (Equations (1) to (3)) [26]. These equations depend on the scattering factor (S), reflectance (R), and the molar absorption coefficient (K). The produced values of CuO and CuFeO_2_ determined from these equations are well-matched with the previous literature [27,28].

(1) $$K={\left(1-R\right)}^{2}$$

(2) $$F\left(R\right)=\frac{K}{S}$$

(3) S=2R

### 3.2. The Optoelectronic Electrochemical Study

#### 3.2.1. The Photoelectrochemical Measurements

**Figure 5:**
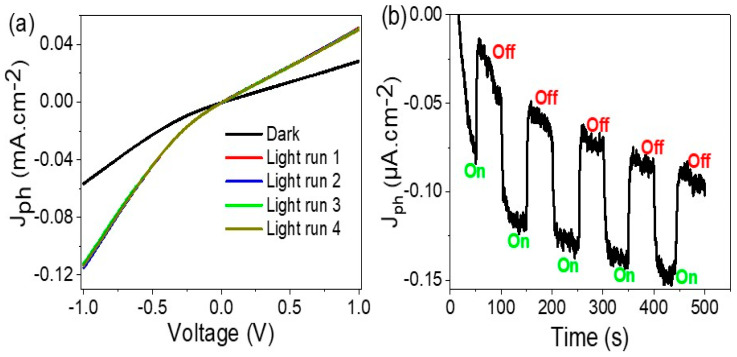
(**a**) The relation of voltage and current density and (**b**) the effect of chopped light illumination on the prepared CuFeO_2_/CuO/Cu optoelectronic device at 100 mW·cm^−^^2^ (solar simulator Xenon lamp).

The photoelectrochemical measurements of the prepared CuFeO_2_/CuO/Cu optoelectronic device were measured in dark and under light, as shown in Figure 5a, while the chopped on/off current was measured with time, as shown in Figure 5b. The measurements were carried out using an electrochemical workstation CHI from −1.0 to +1.0 voltage, under a sweep rate of 100 mV·s^−^^1^. The measurements were carried out under a solar simulator Xenon lamp (100 mW·cm^−^^2^). From the dark and light behavior, the prepared optoelectronic device had great responsivity and sensitivity to the light, in which the J_ph_ value increased from −0.11 to 0.05 mA·cm^−^^2^, in the potential range from −1.0 to +1.0, respectively. The dark current (J_O_) had small values in comparison to the J_ph_ values, in which the J_O_ values changed from −0.055 to 0.028 mA·cm^−^^2^, in the potential range from −1.0 to +1.0, respectively. The small J_O_ values are related to the semiconductor nature of the electrode [29]. The great enhancement in the light confirms the high effect of incident light on the optoelectronic device. The sensitivity occurred through the motivation of the incident light at the active sites of the optoelectronic device, CuFeO_2_/CuO, in which electrons transferred from the valency band to the conduction band in both CuO and CuFeO_2_ materials. This created an electron-hole pair phenomena through the nanomaterials. In addition to that, the Cu plates act as current collectors with high electrical conductivity. Then, under the accumulation of electrons, an electronic cloud can form on the surface of the optoelectronic materials. This cloud can appear as a J_ph_ value through the optoelectronic device, in which the hug of this cloud represents the sensitivity of the prepared optoelectronic device for the incident light. After measuring four runs under light, the optoelectronic device had the same behavior; this confirms the great reproducibility of the prepared optoelectronic device.

The chopped on/off light illumination on the prepared electrode is mentioned in Figure 5b at 0.1 V, which confirms the behavior seen in Figure 5a. Under dark conditions, the J_ph_ had very small values, while under illumination, there was a sudden increase in the J_ph_ values. The chopped behavior confirms the fast change and high responsivity of the optoelectronic device to incident light. From the chopped curve behavior, the produced J_ph_ (on) values increased slowly with time; this indicates more activation of the prepared CuFeO_2_/CuO electrode for H_2_ generation, in which the accumulation of charges over the electrode motivates an additional H_2_ generation reaction.

#### 3.2.2. The Effect of Monochromatic Light on the Optoelectronic Device

**Figure 6:**
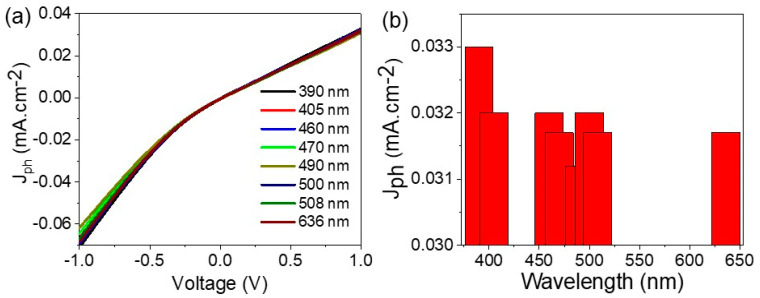
(**a**) The effect of monochromatic light on the prepared optoelectronic device and (**b**) the relation of various wavelengths and the produced current density values at 1.0 V.

The effect of monochromatic light on the prepared optoelectronic device is very important for determination of the device response under different wavelengths [30,31,32,33,34], this effect is studied in the range of 390 to 636 nm, as shown in Figure 6a. Moreover, the J_ph_ values at 1.0 V are shown in Figure 6b. These figures confirm the high sensitivity of the prepared optoelectronic device for light sensing in a wide optical region: UV, Vis, and near IR. The optimum sensitivity is in the UV and the beginning of Vis regions. The J_ph_ value at 390 nm was 0.033 mA·cm^−2^, this value decreased to 0.031 mA·cm^−2^ at 508 nm, and then increased again to 0.0315 mA·cm^−2^ at 636 nm. The sensitivity of the optoelectronic device is related to the different response rate under the light wavelengths [35,36,37]. Under UV light that motivates electron transfer, the electrode has the optimum light response [38,39]. Thus, the prepared optoelectronic device can work as a light detector in UV, Vis, and near-IR regions, with a great advantage being low cost under normal conditions and over large areas. These advantages highlight the potential benefit in using the optoelectronic device for industrial applications.

**Figure 7:**
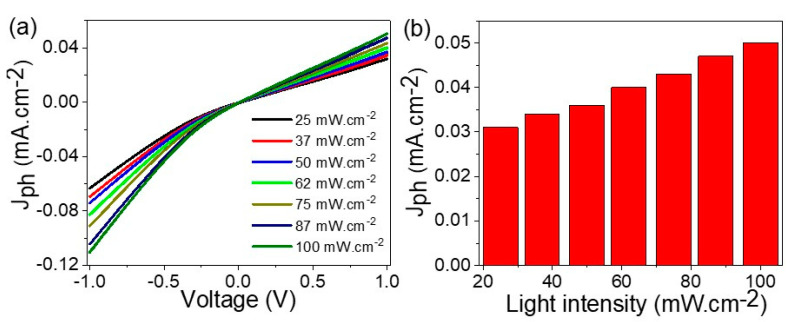
(**a**) The response of the optoelectronic device to different light intensities from 25 to 100 mW·cm^−^^2^ and (**b**) the values of current density with the light intensity at 1.0 V.

The effect of light intensity on the responsivity of the prepared optoelectronic device, CuFeO_2_/CuO/Cu, is shown in Figure 7a. The J_ph_ values with a light intensity at 1.0 V are mentioned in Figure 7b. From these figures, the J_ph_ values increased with an increase in the light intensities from 25 to 100 mW·cm^−2^, in which the J_ph_ values were 0.031 and 0.05 mA·cm^−2^, respectively. The high sensitivity of the optoelectronic device appears well throughout the J_ph_ values under small or significant intensities. This confirms that the large surface of the prepared optoelectronic materials can respond to very small numbers of photons [35]. This is related to the motivation of the CuFeO_2_/CuO/Cu surface with the photon flux, whereby increasing the intensity increases the number of photons, which activates the active sites on the materials. This appears well throughout the responsivity of the nanomaterials with light photons, in which the splitting of energy levels and the generation of electron-hole pairs increases, thereby motivating enhancements in the J_ph_ values [36,37].

#### 3.2.3. Optoelectronic Device Efficiency

**Figure 8:**
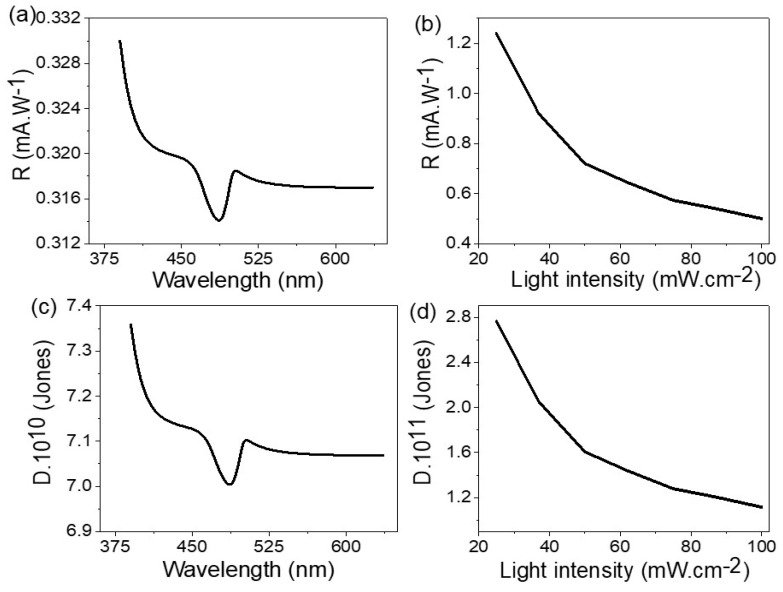
The responsivity value through relation with light (**a**) wavelengths and (**b**) intensity. The detectivity value through the relation with light (**c**) wavelengths and (**d**) intensity.

The efficiency of the optoelectronic device is related to its ability to detect light under different intensities or wavelengths. The R-value [38] is calculated through Equation (4), as this relation represents the ratio of J_ph_-J_O_ and the power intensity, and the relation between the R and wavelengths is shown in Figure 8a. The R values of the fabricated optoelectronic device have an optimum value of 0.33 mA·W^−^^1^ at 390 nm, then this value decreases with an increase in the wavelengths. In the same manner, the relation between the R and light intensity is mentioned in Figure 8b. From this figure, the R-value has an optimum value of 1.22 mA·W^−^^1^ at 25 mW·cm^−^^2^. The determination of D values for the optoelectronic device is determined through the relation mentioned in Equation (5). This relation depends on the R; moreover, it depends on surface area (A) and electron charge (e). The optimum D value is 7.36 *×* 10^10^ Jones at 390 nm. This relation can be calculated through the light intensity, as shown in Figure 8d, where the optimum value is 2.97 *×* 10^11^ Jones obtained at 25 mW·cm^−^^2^, and then with an increase in the light intensity power, the D value for the prepared optoelectronic device decreases.

(4) $$R=\frac{J_{\mathrm{ph}}-{\;J}_{d}}{P}$$

(5) $$D=R\;\sqrt{A/2{\;e\;J}_{d}\;}$$

**Figure 9:**
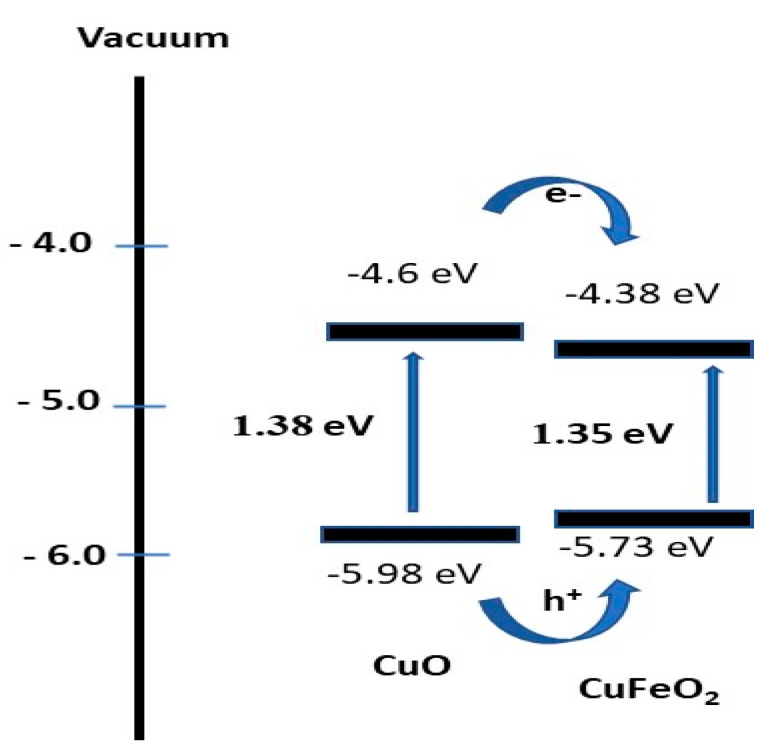
The schematic diagram for the optoelectronic mechanism of CuFeO_2_/CuO/Cu electrode.

Both R and D values prove that the prepared CuFeO_2_/CuO/Cu optoelectronic device can detect the optical light in broad light regions, such as UV, Vis, and near-IR regions. These properties confirm the high activity of CuFeO_2_/CuO/Cu optoelectronic devices for light detection, especially at the beginning of the Vis region. A schematic mechanism for the electron and hole transfer is mentioned in Figure 9. Under light irradiation, both CuO and CuFeO_2_ respond to incidence photons, in which the electron splitting levels are produced [39,40]. The difference in the energy level (conducting band) of CuFeO_2_ and CuO motivates the electron transfer from CuO to CuFeO_2_, and then these charges accumulate over CuFeO_2_. Through measurements, the optoelectronic response appears through the J_ph_ value that represents the accumulation of charges over CuFeO_2_. To confirm the high optical property for the prepared optoelectronic device, a comparison is mentioned in Table 1 between the produced results and the previous literature.

## 4. Conclusions

A CuFeO_2_/CuO/Cu composite was prepared through the direct combustion of Cu foil coated with Fe_2_O_3_ nanomaterials at 500 °C for 10 min in ambient air. From the XPS analyses, the peaks at 952.09 and 931 eV corresponded to the Cu _2p1/2_ and Cu _2p3/2_ spin-orbital components, while Fe 2p XPS demonstrated well-resolved Fe _2p3/2_ and Fe _2p1/2_ peaks at 712 and 726 eV, as confirmed by earlier studies. O 1s XPS spectra displayed a single peak at 529.08 eV. An enhancement in the produced optical bandgap was located after the formation of CuFeO_2_, in which the bandgap values were 1.35 and 1.38 eV for CuO and CuFeO_2_, respectively. The effect of the monochromatic wavelength study was carried out, where the optimum J_ph_ value was 0.033 mA·cm^−2^ at 390 nm. The effect of light intensity was carried out, in which the optimum J_ph_ value was 100 mW·cm^−2^ at 100 mW·cm^−2^. The photoresponsivity (R) and detectivity (D) values were 0.33 mA·W^−1^ and 7.36 × 10^10^ Jones, respectively, at 390 nm. Soon, our team will work to design a prototype of this optoelectronic device that can sense light in a broad optical region that can be applied in the industrial field.

## Figures and Tables

**Table 1:** Comparison of the produced results with the previous literature.

Structure	Wavelength (nm)	Bais (V)	R (mAW^−1^)
GO/Cu_2_O [31]	300	2	0.5 × 10^−3^
CuO nanowires [13]	390	5	-
ZnO/Cu_2_O [15]	350	2	4 × 10^−3^
ZnO-CuO [32]	405	1	3 × 10^−3^
CuO/Si Nanowire [14]	405	0.2	3.8 × 10^−3^
TiN/TiO_2_ [33]	550	5	-
Se/TiO_2_ [34]	450	1	5 × 10^−3^
TiO_2_-PANI [35]	320	0	3 × 10^−3^
TiO_2_/NiO [36]	350	0	0.4 × 10^−3^
Graphene/GaN [37]	365	7	3 × 10^−3^
ZnO /RGO [38]	350	5	1.3 × 10^−3^
CuFeO_2_/CuO/Cu (this work)	390	1	0.33

## Data Availability

Not applicable.

## References

[1] Zhong F., Wang H., Wang Z., Wang Y., He T., Wu P., Peng M., Wang H., Xu T., Wang F. (2020). Recent progress and challenges on two-dimensional material photodetectors from the perspective of advanced characterization technologies. Nano Res..

[2] Liu C., Guo J., Yu L., Li J., Zhang M., Li H., Shi Y., Dai D. (2021). Silicon/2D-material photodetectors: From near-infrared to mid-infrared. Light. Sci. Appl..

[3] Maiti R., Patil C., Saadi M.A.S.R., Xie T., Azadani J.G., Uluutku B., Amin R., Briggs A.F., Miscuglio M., Van Thourhout D. (2020). Strain-engineered high-responsivity MoTe2 photodetector for silicon photonic integrated circuits. Nat. Photonics.

[4] Li Y., An N., Lu Z., Wang Y., Chang B., Tan T., Guo X., Xu X., He J., Xia H. (2022). Nonlinear co-generation of graphene plasmons for optoelectronic logic operations. Nat. Commun..

[5] Lee S., Peng R., Wu C., Li M. (2022). Programmable black phosphorus image sensor for broadband optoelectronic edge computing. Nat. Commun..

[6] Ding H., Lv G., Cai X., Chen J., Cheng Z., Peng Y., Tang G., Shi Z., Xie Y., Fu X. (2022). An Optoelectronic thermometer based on microscale infrared-to-visible conversion devices. Light. Sci. Appl..

[7] Yu X., Marks T.J., Facchetti A. (2016). Metal oxides for optoelectronic applications. Nat. Mater..

[8] Rabia M., Mohamed S.H., Zhao H., Shaban M., Lei Y., Ahmed A.M. (2019). Correction to: TiO_2_/TiOxNY hollow mushrooms-like nanocomposite photoanode for hydrogen electrogeneration. J. Porous Mater..

[9] Almohammedi A., Shaban M., Mostafa H., Rabia M. (2021). Nanoporous TiN/TiO_2_/Alumina Membrane for Photoelectrochemical Hydrogen Production from Sewage Water. Nanomaterials.

[10] Elsayed A.M., Shaban M., Aly A.H., Ahmed A.M., Rabia M. (2021). Preparation and characterization of a high-efficiency photoelectric detector composed of hexagonal Al_2_O_3_/TiO_2_/TiN/Au nanoporous array. Mater. Sci. Semicond. Process..

[11] Hadia N.M.A., Abdelazeez A.A.A., Alzaid M., Shaban M., Mohamed S.H., Hoex B., Hajjiah A., Rabia M. (2022). Converting Sewage Water into H_2_ Fuel Gas Using Cu/CuO Nanoporous Photocatalytic Electrodes. Materials.

[12] Abdelazeez A.A.A., Hadia N.M.A., Alzaid M., Shaban M., Mourad A.-H.I., Fernández S., Rabia M. (2022). Development of CuO nanoporous material as a highly efficient optoelectronic device. Appl. Phys. A Mater. Sci. Process..

[13] Wang S.B., Hsiao C.H., Chang S.J., Lam K.T., Wen K.H., Hung S.C., Young S.-J., Huang B.R. (2011). A CuO nanowire infrared photodetector. Sens. Actuators A Phys..

[14] Hong Q., Cao Y., Xu J., Lu H., He J., Sun J.-L. (2014). Self-Powered Ultrafast Broadband Photodetector Based on p–n Heterojunctions of CuO/Si Nanowire Array. ACS Appl. Mater. Interfaces.

[15] Bai Z., Zhang Y. (2016). Self-powered UV–visible photodetectors based on ZnO/Cu_2_O nanowire/electrolyte heterojunctions. J. Alloys Compd..

[16] Ma S., Yu L., Cao L., Li C., Yin M., Fan X. (2020). Resistive-type UV–visible photodetector based on CdS NWs /ZnO nanowalls heterostructure fabricated using in-situ synthesis method. J. Alloys Compd..

[17] Nien Y.-T., Chen Y.-Z., Hsu Y.-R., Ye H.-J. (2021). Enhanced antibacterial effect of CuFeO2 ceramic powders by glycine combustion process and visible light irradiation. Mater. Chem. Phys..

[18] Wheatley R., Roble M., Gence L., Acuña C., Rojas-Aedo R., Hidalgo-Rojas D., La Cerda D.G.-D., Vojkovic S., Seifert B., Wallentowitz S. (2020). Structural, optoelectronic and photo-thermoelectric properties of crystalline alloy CuAlxFe1-xO_2_ delafossite oxide materials. J. Alloys Compd..

[19] Prévot M.S., Guijarro N., Sivula K. (2015). Enhancing the Performance of a Robust Sol-Gel-Processed p-Type Delafossite CuFeO_2_Photocathode for Solar Water Reduction. ChemSusChem.

[20] Omeiri S., Bellal B., Bouguelia A., Bessekhouad Y., Trari M. (2009). Electrochemical and photoelectrochemical characterization of CuFeO_2_ single crystal. J. Solid State Electrochem..

[21] Roble M., Rojas S., Wheatley R., Wallentowitz S., Cabrera A., Diaz-Droguett D. (2019). Hydrothermal improvement for 3R-CuFeO_2_ delafossite growth by control of mineralizer and reaction atmosphere. J. Solid State Chem..

[22] Chen H.-Y., Fu J.-R. (2014). Delafossite–CuFeO_2_ thin films prepared by atmospheric pressure plasma annealing. Mater. Lett..

[23] Read C.G., Park Y., Choi K.S. (2012). Electrochemical synthesis of p-type CuFeO_2_ electrodes for use in a photoelectrochemical cell. J. Phys. Chem. Lett..

[24] Crespo C.T. (2018). Potentiality of CuFeO_2_-delafossite as a solar energy converter. Sol. Energy.

[25] Deng Z., Fang X., Wu S., Dong W., Shao J., Wang S., Lei M. (2014). The morphologies and optoelectronic properties of delafossite CuFeO_2_ thin films prepared by PEG assisted sol–gel method. J. Sol-Gel Sci. Technol..

[26] Sivakumar S., Manikandan E. (2019). Enhanced structural, optical, electrochemical and magnetic behavior on manganese doped tin oxide nanoparticles via chemical precipitation method. J. Mater. Sci. Mater. Electron..

[27] Ravichandran A.T., Dhanabalan K., Vasuhi A., Chandramohan R., Mantha S. (2014). Morphology, Bandgap, and Grain Size Tailoring in Cu_2_O Thin Film by SILAR Method. IEEE Trans. Nanotechnol..

[28] Patil V., Jundale D., Pawar S., Chougule M., Godse P., Patil S., Raut B., Sen S. (2011). Nanocrystalline CuO Thin Films for H_2_S Monitoring: Microstructural and Optoelectronic Characterization. J. Sens. Technol..

[29] Elsayed A.M., Rabia M., Shaban M., Aly A.H., Ahmed A.M. (2021). Preparation of hexagonal nanoporous Al_2_O_3_/TiO_2_/TiN as a novel photodetector with high efficiency. Sci. Rep..

[30] Mohamed F., Rabia M., Shaban M. (2020). Synthesis and characterization of biogenic iron oxides of different nanomorphologies from pomegranate peels for efficient solar hydrogen production. J. Mater. Res. Technol..

[31] Rabia M., Shaban M., Adel A., Abdel-Khaliek A.A. (2019). Effect of plasmonic au nanoparticles on the photoactivity of polyaniline/indium tin oxide electrodes for water splitting. Environ. Prog. Sustain. Energy.

[32] Rabia M., Shaban M., Jibali B.M., Abdelkhaliek A.A. (2020). Effect of Annealing Temperature on the Photoactivity of ITO/VO_2_(M)/Au Film Electrodes for Water Splitting. J. Nanosci. Nanotechnol..

[33] Liu Z., Li F., Li S., Hu C., Wang W., Wang F., Lin F., Wang H. (2015). Fabrication of UV Photodetector on TiO_2_/Diamond Film. Sci. Rep..

[34] Gamal A., Alruqi M., Rabia M. (2022). CsPbI_3_ Lead and CsSnI_3_ Lead-Free Perovskite Materials for Solar Cell Device. Int. J. Energy Res..

[35] Khalafalla M.A.H., Hadia N.M.A., Elsayed A.M., Alruqi M., El Malti W., Shaban M., Rabia M. (2022). ATO/Polyaniline/PbS Nanocomposite as Highly Efficient Photoelectrode for Hydrogen Production from Wastewater with Theoretical Study for the Water Splitting. Adsorp. Sci. Technol..

[36] Algadi H., Mahata C., Woo J., Lee M., Kim M., Lee T. (2019). Enhanced Photoresponsivity of All-Inorganic (CsPbBr_3_) Perovskite Nanosheets Photodetector with Carbon Nanodots (CDs). Electronics.

[37] Algadi H., Umar A., Albargi H., Alsuwian T., Baskoutas S. (2021). Carbon Nanodots as a Potential Transport Layer for Boosting Performance of All-Inorganic Perovskite Nanocrystals-Based Photodetector. Crystals.

[38] Jia R., Zhao D., Gao N., Liu D. (2017). Polarization Enhanced Charge Transfer: Dual-Band GaN-Based Plasmonic Photodetector. Sci. Rep..

[39] Albargi H., Umar A., Shkir M. (2021). Enhanced photoresponsivity of anatase titanium dioxide (TiO_2_)/nitrogen-doped graphene quantum dots (N-GQDs) heterojunction-based photodetector. Adv. Compos. Hybrid Mater..

[40] Algadi H., Mahata C., Kim S., Dalapati G.K. (2020). Improvement of Photoresponse Properties of Self-Powered ITO/InP Schottky Junction Photodetector by Interfacial ZnO Passivation. J. Electron. Mater..

